# The complete plastome and phylogenetic analysis of *Commelina benghalensis* L.1753 (Commelinaceae)

**DOI:** 10.1080/23802359.2024.2347508

**Published:** 2024-05-08

**Authors:** Liqiang Wang, Shuming Zhang, Hongqin Li, Shu Wang

**Affiliations:** College of Pharmacy, Heze University, Heze, Shandong Province, P. R. China

**Keywords:** *Commelina benghalensis*, next-generation sequencing, genome assembly, phylogeny

## Abstract

*Commelina benghalensis* L. 1753, a member of the Commelinaceae family, holds significant medicinal and culinary value. This study represents the first documentation of the sequencing and assembly of the entire plastome of *C. benghalensis*. The genome spans a total length of 160,663 bp, exhibiting a conventional quadripartite architecture that comprises a large single-copy (LSC) region (87,750 bp), a small single-copy (SSC) region (18,417 bp), and two inverted repeats (IR) regions (both 27,248 bp). In its entirety, the *C. benghalensis* plastome encompasses 129 genes (with 108 being unique), incorporating 77 individual protein-coding genes, 37 unique tRNA genes, and four unique rRNA genes. Phylogenetic analysis revealed a close resemblance between *C. benghalensis* and *C. communis*. The sequencing of this plastome stands to expedite the development of molecular markers and significantly contribute to genetic assays involving this distinctive plant.

## Introduction

*Commelina*, the largest genus in the Commelinaceae family, comprises around 170 species known as dayflowers due to their short-lived blooms (Pellegrini and Forzza 2017). These perennial or annual herbaceous plants exhibit zygomorphic flowers and distinctive spathes encircling their flower stalks. Typically displaying blue or purple flowers with three unequal petals, they also feature sword-shaped or ovate leaves, spike-like inflorescences, and capsule fruits. Certain species like *Commelina diffusa* Burm.f. 1768 and *Commelina benghalensis* L. 1753 are utilized for medicinal and edible purposes (Wang et al. 2023).

*Commelina benghalensis*, a widely distributed folkloric medicinal plant in Asia, Africa, and South America, has long been recognized for its therapeutic properties. This plant is a rich source of diverse bioactive compounds, including alkaloids, volatile oils, wax, vitamin C, and high levels of lutein and β-carotene (Kansagara and Pandya 2019). Pharmacologically, *C. benghalensis* exhibits a broad spectrum of activities, including laxative, anti-inflammatory, antimicrobial, anticancer, sedative, analgesic, hepatoprotective, antidepressant, antiviral, antioxidant, antidiarrheal, demulcent, emollient, diuretic, and febrifuge properties. In traditional medicine, it has been used to treat a variety of ailments such as pain, constipation, headache, leprosy, fever, snake bites, jaundice, psychosis, epilepsy, nose blockage, insanity, and exophthalmia (Pranabesh et al. 2019). In Chinese traditional medicine, *C. benghalensis* is valued for its diuretic, febrifuge, laxative, and anti-inflammatory effects (Hasan et al. 2009). While numerous studies have focused on the pharmacological activities, compound isolation, and toxicity of this plant, there is still a limited amount of molecular research conducted on it.

To date, the plastome of *C. communis* has been reported (Cui and Liang 2019) in the *Commelina* genus (MK863371). Another version of the plastome of *C. communis* (MW617984) and the plastome of *C. caroliniana* has also been deposited in the GenBank (OR936140). Understanding genetic diversity is crucial for *Commelina* conservation and elucidating its evolution. For example, genetic analysis was conducted on *C. communis* f. *ciliata* using microsatellite markers to understand its genetic diversity and population structure (Katsuhara et al. 2019). Additionally, the Angiosperms353 probe set was employed to capture target sequences from the nucleus, plastids, and publicly available transcriptomes and complete plastomes, resulting in the extraction of additional sequences. Three large datasets were generated to analyze the phylogenetic relationships within the order Commelinales (Zuntini et al. 2021). To contribute more genetic data and assess the phylogenetic position of *C. benghalensis* within the *Commelina* genus, we sequenced and characterized the complete plastome of *C. benghalensis*, aiming to support further evolutionary research.

## Materials

**Figure 1.**
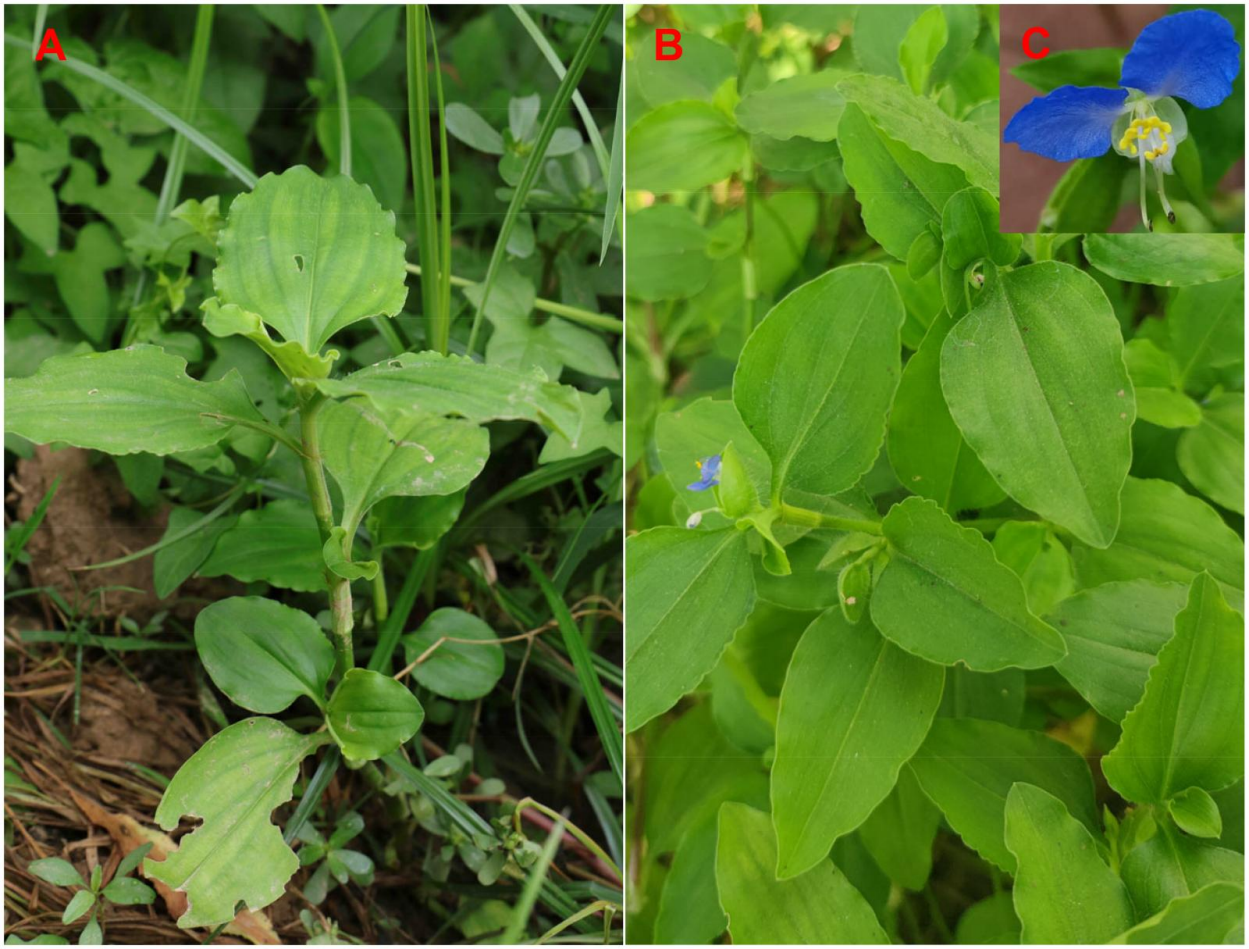
Habitat photos of *Commelina benghalensis*. The photos were taken by Liqiang Wang. Panel A shows a panorama, Panel B shows a detail, and Panel C shows the flowers. The plant's coordinates are N 35° 16′ 23.62″, E 115° 27′ 36.01″. It's a perennial herb with mostly creeping stems reaching up to 70 cm in height and ovate leaves measuring 3-7 cm long. The flowers, enclosed in funnel-shaped bracts, bloom in the summer to autumn season. This plant thrives in tropical and subtropical wetlands, from near sea level to 2300 meters in altitude.

The *C. benghalensis* samples (Figure 1) were collected from Peony District, Heze City, Shandong Province, China (35° 16′ 23.62′’ N, 115° 27′ 36.01′’ E). The specimen was deposited at Heze University Herbarium (contact person: Hongqin Li, 463056627@qq.com) under specimen number HZ20220806. The fresh leaves were used to extract total genomic DNA by using the plant genomic DNA kit (Tiangen Biotech, Beijing, China)

## Methods

Total genomic DNA extracts were fragmented into about 300 bp short-insert fragments for library construction and were sequenced 2 × 150 bp paired-end reads on Illumina NovaSeq 6000 technology platforms at Wuhan Benagen Technology Company Limited (Wuhan, China). The filtering of raw reads was performed using Trimmomatic 0.35 (Bolger et al. 2014), *e.g.* removing adapters and low-quality bases. Then, about 34 GB of clean reads were assembled by using GetOrganelle v1.7.1 (Jin et al. 2020). The finished plastome was annotated by using CPGAVAS2 (Shi et al. 2019), and then manually adjusted by Apollo (Pontius 2018). The sequencing depth of the genome was calculated by BWA (Li and Durbin 2009) coupled with samtools (Li et al. 2009). Finally, the annotated plastome was submitted to GenBank using Bankit (The accession number is OQ2656460). The CPGview (http://www.1kmpg.cn/Cpgview/) was used to illustrate the circular genome map of the new plastome (Liu et al. 2023).

To determine the phylogenetic relationships of *C. benghalensis*, 30 of the Commelinaceae species plastome with the highest similarity to that of *C. benghalensis* were selected based on blast results of whole plastome sequences in the GenBank. Two outgroups of *Acorus calamus* (AJ879453) and *Acorus tatarinowii* (MN536753) were downloaded from GenBank. The 23 entire plastome sequences were aligned using the MAFFT (version 7) software with default parameters (Katoh and Standley 2016). Then, a maximum-likelihood (ML) phylogenetic tree was constructed based on the Best-fit model of GTR + F + I + R4 according to BIC by IQ-TREE v2.0 (Nguyen et al. 2015) with 1000 bootstrap replicates.

## Results

**Figure 2.**
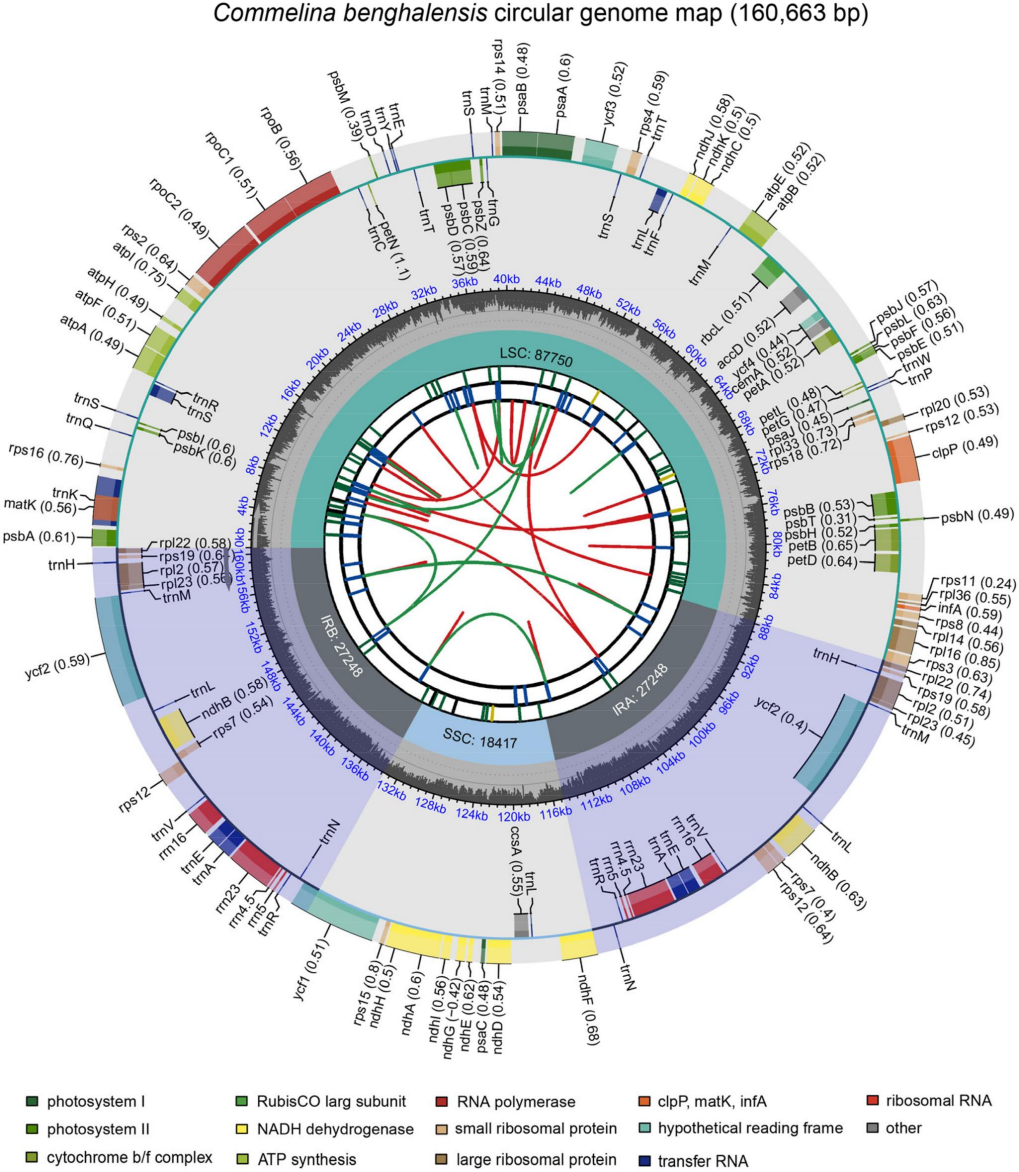
Schematic map of overall features of the *commelina benghalensis* plastome. From the inside out, the first track shows dispersed repeats, which consist of direct and palindromic repeats connected by red and green arcs. The second track shows long tandem repeats as short blue bars, while the third track shows short tandem repeats or microsatellite sequences as short bars with different colors. The fourth track shows the small single-copy, inverted repeat, and large single-copy regions. The fifth track displays the GC content along the genome, and the sixth track shows genes color-coded by their functional classification and transcription direction. The species name is shown in the top left corner, and the bottom left corner provides the functional classification of the genes.

The plastome of the *C. benghalensis* deciphered in this study is a circular DNA molecule with a total length of 160,663 bp. The reliability of genome assembly was strongly supported by the results of the mapping experiment (Figure S1 A, B). The maximum sequencing depth, average sequencing depth, and minimum sequencing depth were 7,992×, 2789.5× and 52×. The comparison of reads mapped to genomes at the loci with minimum sequencing depth is shown in Figure S1 B. The genome has a conservative quadripartite structure, including a large-single copy (LSC) region, a small-single copy (SSC) region, and a pair of inverted repeats (IR) regions, with a length of 87,750 bp, 18,417 bp, and 27,248 bp, respectively. The GC content of the whole genome is 35.83%, which is lower than that of IR regions (42.24%) and higher than that of the LSC (33.13%) and SSC regions (29.77%) (Table S1). The plastome contains 129 genes (108 unique genes), including 77 distinct proteins, 27 distinct tRNAs, and four distinct rRNA genes (Figure 2, Table S2). Eight protein-coding genes (*atp*F, *ndh*A, *ndh*B, *pet*B, *pet*D, *rpl*16, *rpl*2, *rpo*C1) contain one intron, and three genes (*clp*P, *rps*12, *ycf*3) contain two introns (Table S2). The genome contains 11 unique cis-splicing genes (*atp*F, *clp*P, *ndh*A, *ndh*B (×2), *pet*B, *pet*D, *rpl*2 (×2), *rpl*16, *rpo*C2, *rpo*C1, *ycf*3) (Figure S2 A) and one unique trans-splicing gene rps12 (Figure S2 B). Five unique tRNA genes (*trn*A-UGC (×2), *trn*I-GAU (×2), *trn*K-UUU, *trn*L-UAA, *trn*S-CGA) contain one intron.

**Figure 3.**
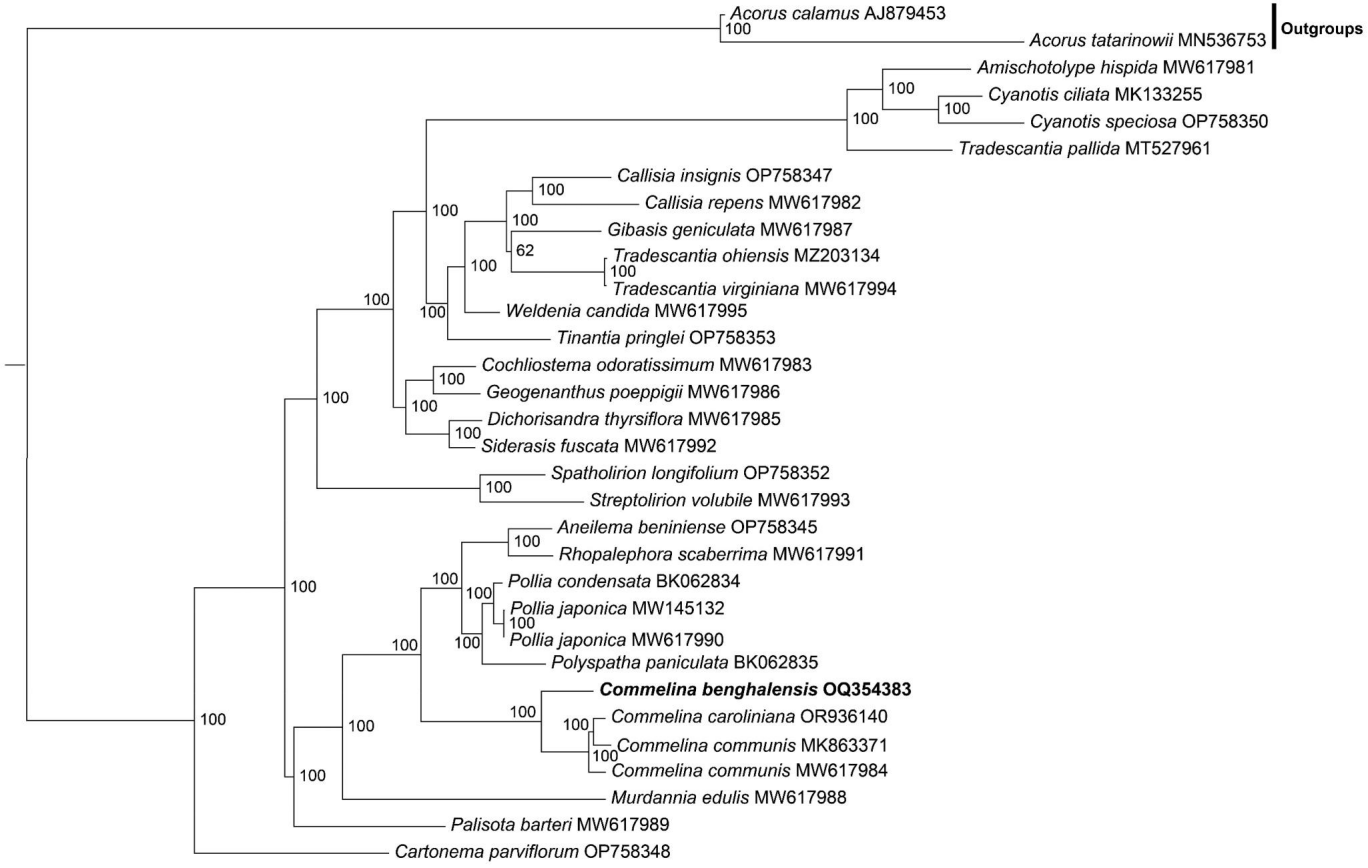
The phylogenetic tree of 30 Commelinaceae species and two outgroups. The tree was constructed with complete plastome sequences present in all 23 species using the maximum likelihood method. The 23 species were *Acorus calamus* (AJ879453, outgroup) (Goremykin et al. 2005), *Acorus tatarinowii* (MN536753, outgroup) (Ma et al. 2020), *Amischotolype hispida* (MW617981) (Jung et al. 2021), *Cyanotis ciliata* (MK133255), *Cyanotis speciosa* (OP758350), *Tradescantia pallida* (MT527961) (Gao et al. 2020), *Callisia insignis* (OP758347), *Callisia repens* (MW617982) (Jung et al. 2021), *Gibasis geniculate* (MW617987) (Jung et al. 2021), *Tradescantia ohiensis* (MZ203134) (Liu et al. 2021), *Tradescantia virginiana* (MW617994) (Jung et al. 2021), *Weldenia candida* (MW617995) (Saha and Jha 2019), *Tinantia pringlei* (OP758353), *Cochliostema odoratissimum* (MW617983) (Jung et al. 2021), *Geogenanthus poeppigii* (MW617986) (Jung et al. 2021), *Dichorisandra thyrsiflora* (MW617985) (Jung et al. 2021), *Siderasis fuscata* (MW617992) (Jung et al. 2021), *Spatholirion longifolium* (OP758352), *Streptolirion volubile* (MW617993) (Jung et al. 2021), *Aneilema beniniense* (OP758345), *Rhopalephora scaberrima* (MW617991) (Jung et al. 2021), *Pollia condensata* (BK062834), *Pollia japonica* (MW145132) (Gu and Ma 2021), *Pollia japonica* (MW617990), *Polyspatha paniculata* (BK062835), *Commelina benghalensis* (OQ354383, this study), *Commelina caroliniana* (OR936140), *Commelina communis* (MK863371) (Cui and Liang 2019), *Commelina communis* (MW617984), *Murdannia edulis* (MW617988) (Jung et al. 2021), and *Palisota barteri* (MW617989) (Jung et al. 2021), *Cartonema parviflorum* (OP758348). Bootstrap supports were calculated from 1000 replicates. The *Commelina benghalensis* was labeled in bold font in the phylogenetic tree

In order to analyze the phylogenetic position of *C. benghalensis* in the Commelinaceae, we reconstructed the phylogenetic tree of the Commelinaceae species with the whole plastome sequences. Phylogenetic analysis shows that *Commelina* species form a monophyletic clade with a bootstrap value of 100. *Commelina benghalensis* is placed at the base of the *Commelina* genus (Figure 3).

## Discussion and conclusion

We present the first comprehensive plastome analysis of *C. benghalensis*. The plastome exhibits a conservative quadripartite structure spanning 160,663 base pairs and encodes 129 genes. Phylogenetic analysis reveals all *Commelina* species formed a monophyletic clade, supported by a robust bootstrap value of 100.

The length of this plastome exceeds that of *C. communis* by 999 base pairs. However, it contains eight fewer genes than the *C. communis* plastome (Cui and Liang 2019). The evolutionary tree reconstructed using complete plastomes for the species of Commelinaceae is topologically consistent with the tree reconstructed from shared protein-coding gene sequences (Jung et al. 2021). Due to its position at the base of the *Commelina* genus evolutionary tree, *C. benghalensis* may be one of the oldest species in the *Commelina* genus. Plastomes with high conservation have been widely used in species identification and phylogenetic analysis (Szymon et al. 2016). Molecular markers developed from plastomes are potentially valuable for studying the intra- and inter-species genetic structure. Establishing a phylogenetic tree based on plastomes will enhance our understanding of the genetic structure and evolution in *Commelina* species.

In the phylogenetic analysis, *Tradescantia pallida*, *T. ohiensis* and *T. virginiana* do not constitute a monophyletic group. A prevalent issue in phylogenomic data is the inconsistency between gene trees and species trees, which obstructs our efforts to accurately reconstruct and interpret the tree of life (Steenwyk et al. 2023). This incongruence can arise from various biological factors such as incomplete lineage sorting, horizontal gene transfer, hybridization, introgression, recombination, and convergent molecular evolution, resulting in gene phylogenies that diverge from the species tree. Additionally, analytical factors like stochastic, systematic, and treatment errors can also contribute to this inconsistency (Steenwyk et al. 2023). To elucidate the phylogenetic relationship of Commelinaceae members, future research should prioritize the exploration of additional plastomes within this family.

## Supplementary Material

Supplemental Material

## Data Availability

The plastome sequence has been deposited in GenBank (https://www.ncbi.nlm.nih.gov/genbank/) with the accession number OQ354383 (https://www.ncbi.nlm.nih.gov/nuccore/OQ354383). The associated BioProject, Bio-Sample, and SRA numbers are PRJNA928567, SAMN36852649 and SRR23251264. (https://www.ncbi.nlm.nih.gov/sra/?term=SRR12620715).
