# Supplementary material for: The complete plastome and phylogenetic analysis of Commelina benghalensis L.1753 (Commelinaceae)
Source: Mitochondrial DNA B Resour. 2024 May 8;9(5):610–5. doi: 10.1080/23802359.2024.2347508 (PMC11086016; doi:10.1080/23802359.2024.2347508)
Supplement: Supplemental Material [file TMDN_A_2347508_SM5651.docx]

**B**


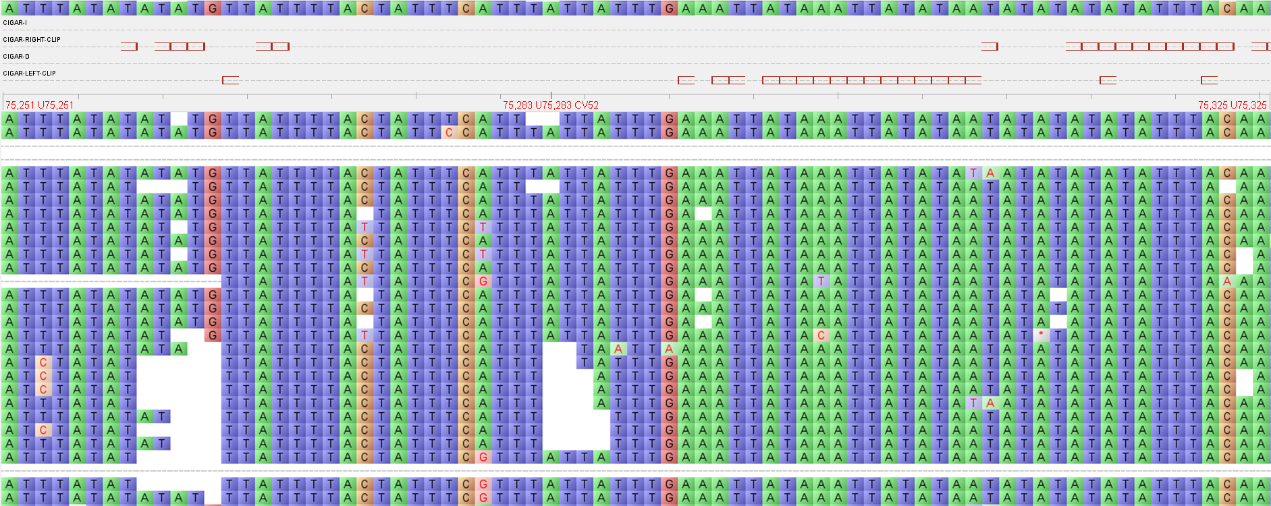


**Figure S1.** The sequencing depth and coverage map results of the assembled *Commelina benghalensis* plastome. (A) The sequencing depth of the whole assembled plastome. (B) The coverage map of the plastome from position 75,251 to 75,325 bp with the minor coverage depth (52×).


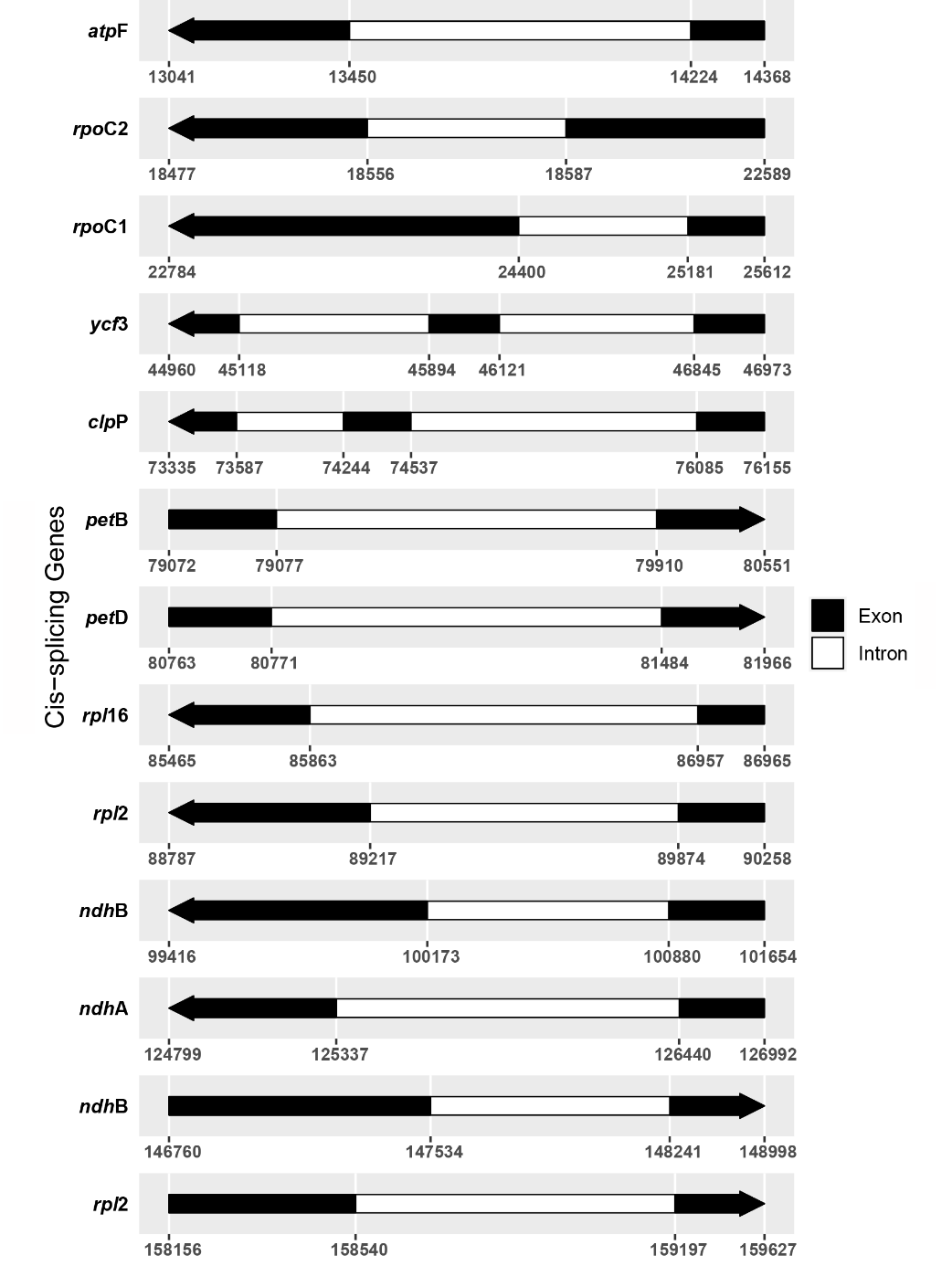


**A**


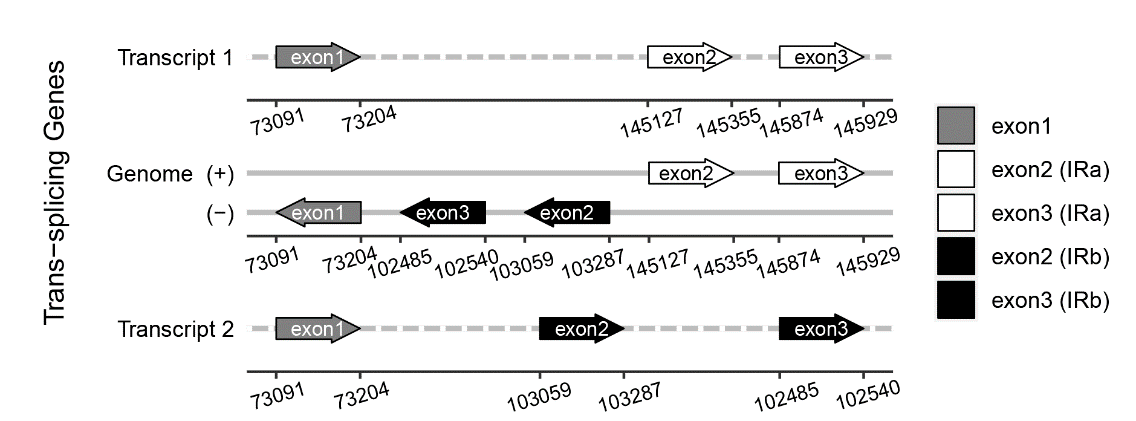


**B**

**Figure S2.** The structures of the genes with introns in the *Commelina benghalensis* plastome. (A) The structures of 13 cis-splicing genes. (B) The structure of the trans-splicing gene *rps*12.

**Table S1.** Summary of plastome feature of *Commelina benghalensis*.

| Region | Code position | Base content (%) | | | | Length (bp) | Percent of whole genome (%) |
| --- | --- | --- | --- | --- | --- | --- | --- |
|  |  | A | C | G | T |  |  |
| LSC |  | 33.16 | 16.95 | 16.18 | 33.71 | 87,750 | 54.62 |
| SSC |  | 34.48 | 15.34 | 14.43 | 35.74 | 18,417 | 11.46 |
| IRa |  | 28.71 | 20.45 | 21.79 | 29.06 | 27,248 | 16.96 |
| IRb |  | 29.06 | 21.79 | 20.45 | 28.71 | 27,248 | 16.96 |
| Whole genome |  | 31.86 | 18.18 | 17.65 | 32.31 | 160,663 | 100.0 |
| Protein-coding sequence | First | 30.71 | 18.48 | 25.70 | 25.11 | 136,977 | 85.26 |
|  | Second | 29.79 | 19.93 | 17.62 | 32.65 |  |  |
|  | Third | 31.50 | 14.48 | 16.55 | 37.48 |  |  |
| rRNA |  | 21.72 | 23.75 | 29.34 | 25.18 | 2,808 | 1.75 |
| tRNA |  | 26.21 | 23.47 | 31.48 | 18.85 | 9,040 | 5.63 |

**Table S2.** Gene content in the plastome of *Commelina benghalensis*.

| Gene function | Gene type | Gene name |
| --- | --- | --- |
| rRNA | rRNA genes | *rrn*4.5S (×2), rrn5S (×2), *rrn*16S (×2), *rrn*23S (×2) |
| tRNA | tRNA genes | *trn*A-UGC (×2)*, *trn*C-GCA, *trn*D-GUC, *trn*E-UUC, *trn*I-GAU (×2)*, *trn*F-GAA, *trn*G-GCC, *trn*H-GUG (×2), *trn*K-UUU*, *trn*L-CAA (×2), *trn*L-UAA*, *trn*L-UAG, *trn*M-CAU (×4), *trn*N-GUU (×2), *trn*P-UGG, *trn*Q-UUG, *trn*R-ACG (×2), *trn*R-UCU, *trn*S-CGA*, *trn*S-GCU, *trn*S-GGA, *trn*S-UGA, *trn*T-GGU, *trn*T-UGU, *trn*V-GAC (×2), *trn*W-CCA, *trn*Y-GUA |
| Selfduplicate | Small subunit of ribosome | *rps*2, *rps*3, *rps*4, *rps*7 (×2), *rps*8, *rps*11, *rps*12 (×2)**, *rps*14, *rps*15, *rps*16, *rps*18, *rps*19 (×2) |
|  | Large subunit of ribosome | *rpl*2 (×2)*, *rpl*14, *rpl*16*, *rpl*20 , *rpl*22 (×2), *rpl*23 (×2), *rpl*33, *rpl*36 |
|  | DNA dependent RNA polymerase | *rpo*B, *rpo*C1*, *rpo*C2 |
| Photosynthesis | Subunits of NADH-dehydrogenase | *ndh*A*, *ndh*B (×2)*, *ndh*C, *ndh*D, *ndh*E, *ndh*F, *ndh*G, *ndh*H, *ndh*I, *ndh*J, *ndh*K |
|  | Subunits of photosystem Ⅰ | psaA, psaB, psaC, psaJ |
|  | Subunits of photosystem Ⅱ | *psb*A, *psb*B, *psb*C, *psb*D, *psb*E, *psb*F, *psb*H, *psb*I, *psb*J, *psb*K, *psb*L, *psb*M, *psb*N, *psb*T, *psb*Z, *ycf*3** |
|  | Subunits of cytochrome b/f complex | *pet*A, *pet*B*, *pet*D*, *pet*G, *pet*L, *pet*N |
|  | Subunits of ATP synthase | *atp*A, *atp*B, *atp*E, *atp*F*, *atp*H, *atp*I |
|  | Large subunit of rubisco | *rbc*L |
| Other genes | Maturase | *mat*K |
|  | Protease | *clp*P** |
|  | Envelope membrane protein | *cem*A |
|  | Subunit of Acetyl-CoA-carboxylase | *acc*D |
|  | c-type cytochrom synthesis gene | *ccs*A |
|  | Translational initiation factor | *inf*A |
| Genes of unknown functions | Open Reading Frame | *ycf*1, *ycf*2 (×2), *ycf*4 |

Note: The numbers in parentheses after the gene name indicate the copy number of this gene in the *C. benghalensis* plastome. The number of asterisks in the upper right corner of the gene name indicates the number of introns in the gene.
